# Supplementary material for: FAM35A associates with REV7 and modulates DNA damage responses of normal and BRCA1‐defective cells
Source: EMBO J. 2018 May 22;37(12):e99543. doi: 10.15252/embj.201899543 (PMC6003645; doi:10.15252/embj.201899543)
Supplement: Supplementary file 1 — Expanded View Figures PDF [file EMBJ-37-e99543-s001.pdf]

Expanded View Figures

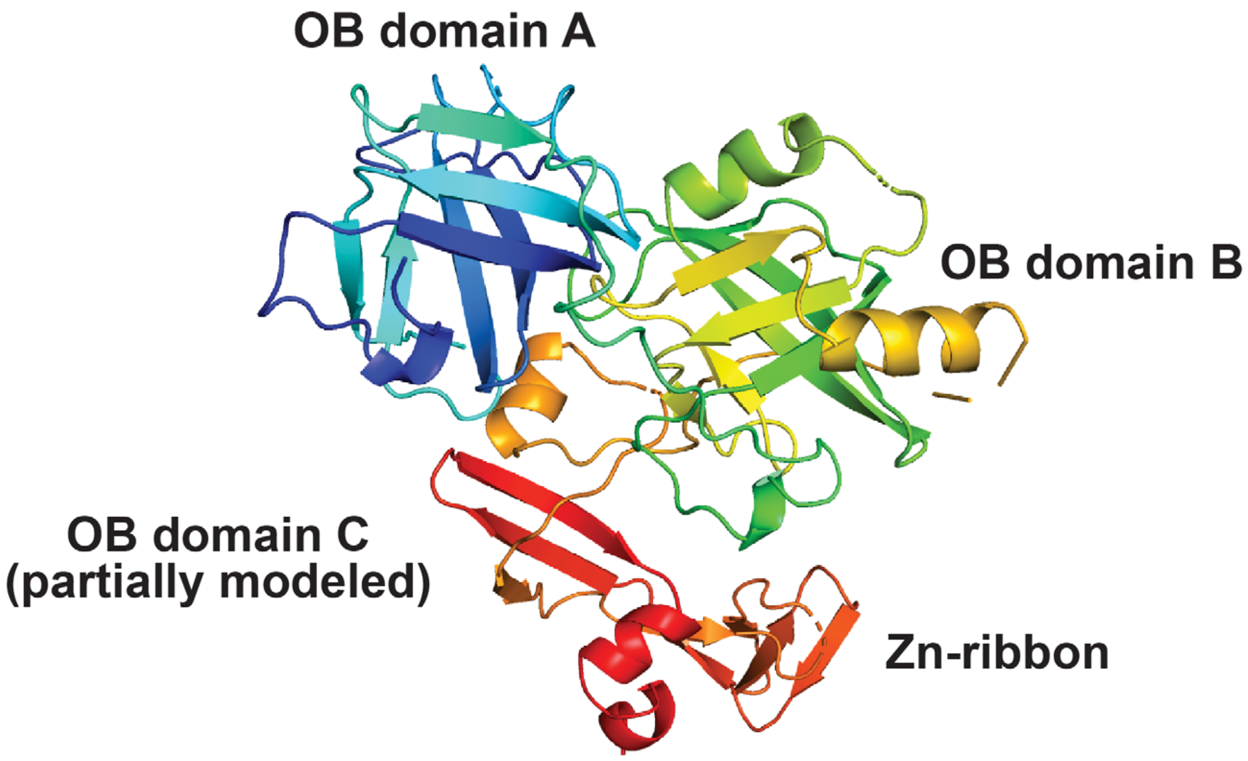

Figure EV1. Partial model of the OB-fold domains of FAM35A.

A view of a model generated by Phyre2 is shown for FAM35A isoform 1 residues 402–904, using template 4GOP (chain C, the RPA 70 kDa subunit from *Ustilago maydis*). The model includes OB folds with five-stranded beta barrels for OB domain A and OB domain B. The first three beta strands of OB domain C are modeled, with the Zn-ribbon that occurs in a loop between OB fold C beta strands 1 and 2 (Fig EV2). The Phyre2 coordinates were rendered in PyMOL using spectrum coloring.

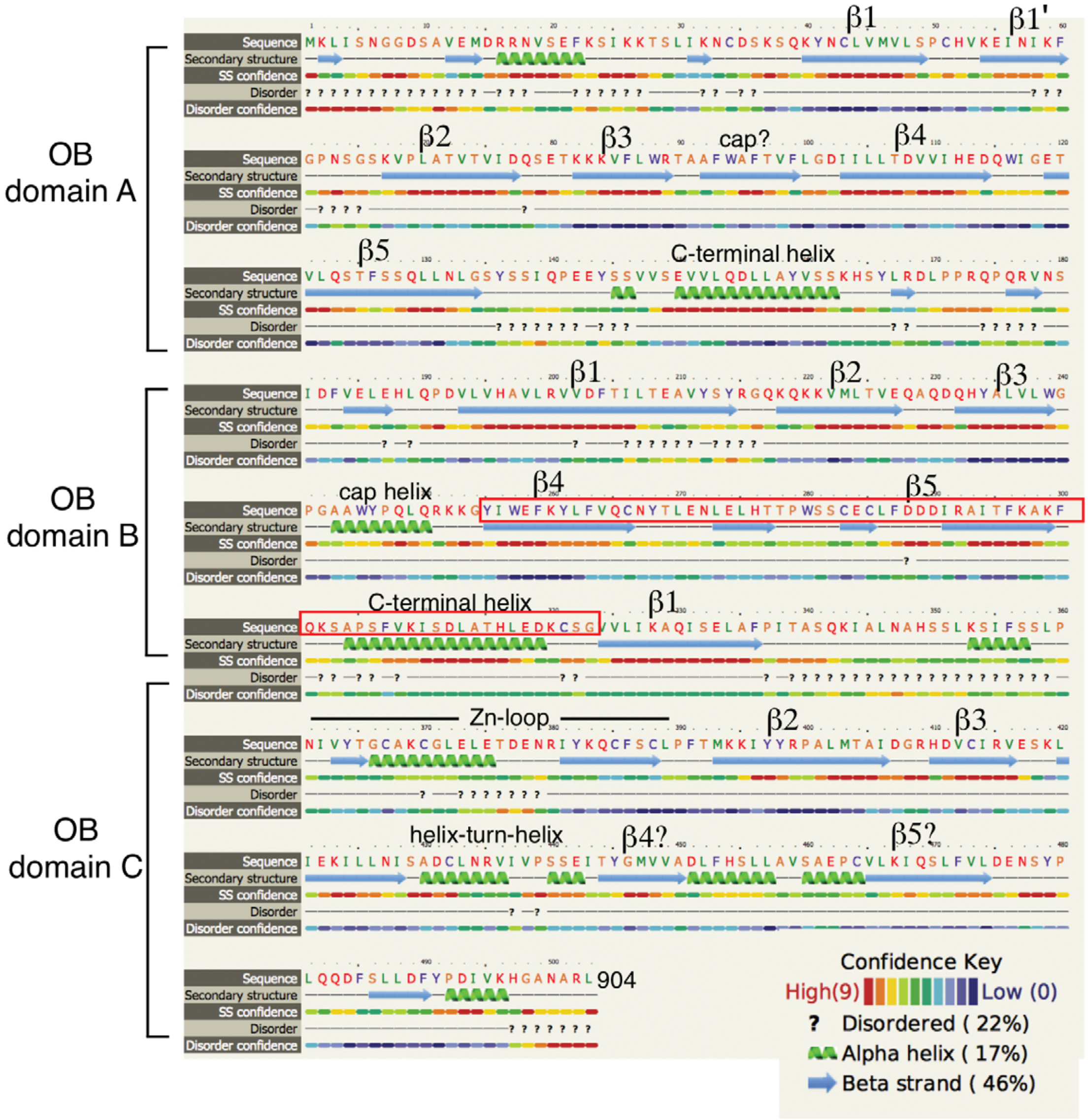

Figure EV2. Secondary structure element assignment for the ordered region of human FAM35A.

The secondary structure assignment is derived from the Phyre2 model of human FAM35A isoform 1 (Fig EV1) and largely coincides with features in the three functional DNA-binding domains (dbdA, B, and C) of RPA1 (PDB: 4GOP, 1FGU), with a few differences. In FAM35A, a C-terminal helix is predicted to be present at the end of both OB folds A and B, unlike in RPA1 where this helix only follows OB domain B. Beta strands 4 and 5 of OB domain C were not modeled by Phyre2 but candidate beta strands are present in appropriate positions, as noted. The exon that is absent in FAM35A isoform 2 results in 69 fewer residues (red box), deleting structural elements of OB domain B.

© 2018 The Authors

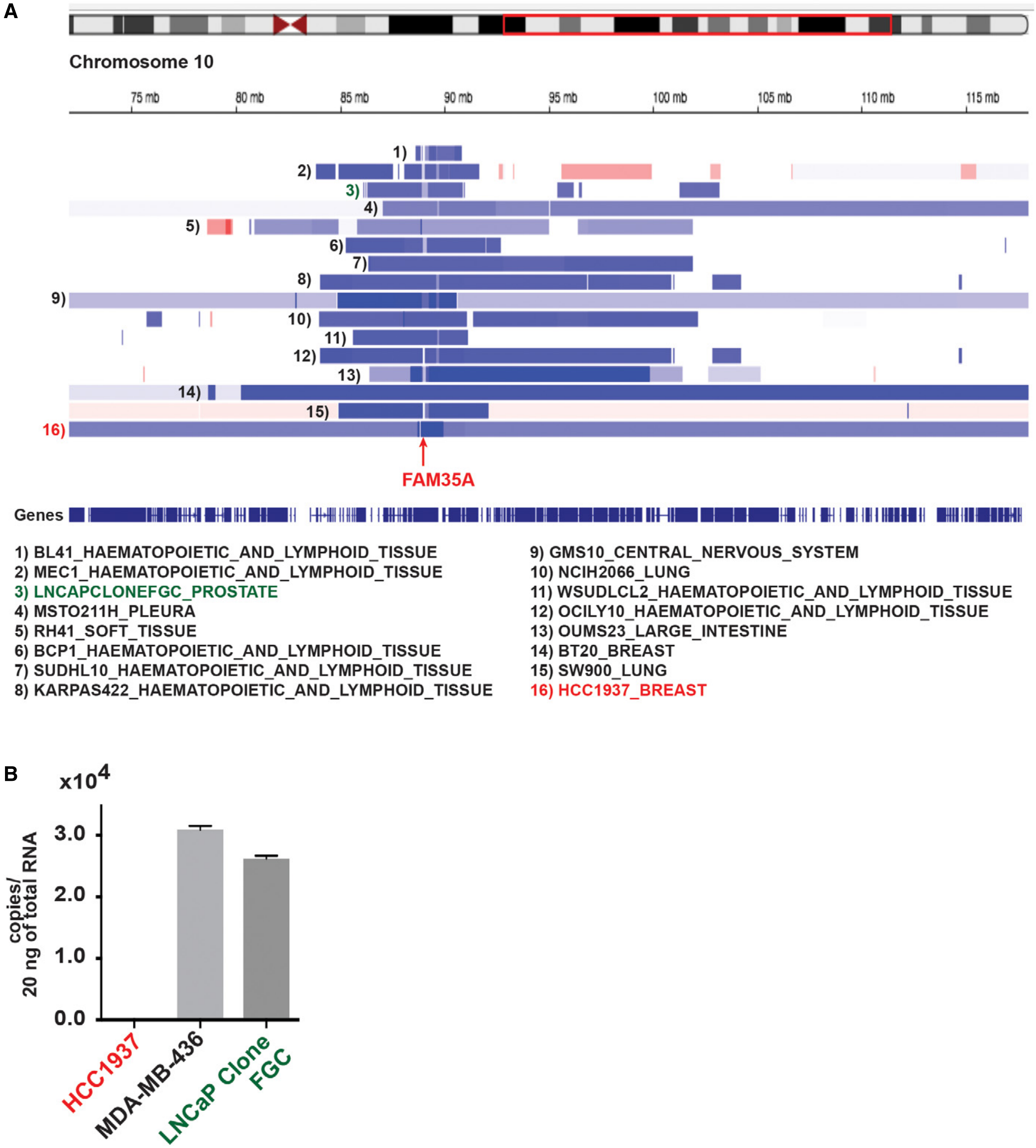

Figure EV3. Potential FAM35A alterations in human cancer cell lines.

A Cell lines annotated in the Cancer Cell Line Encyclopedia with deep deletion of FAM35A. Data accessed from cBioPortal, February 2018. A region of chromosome 10q (red box) is expanded below to show calls for deep deletion (dark blue), shallow deletion (light blue), or amplification (red).

B Quantification of endogenous FAM35A mRNA expression. Endogenous FAM35A mRNA from HCC1937 (*BRCA1* mutant), MDA-MB-436 (*BRCA1* mutant), and LNCaP clone FGC cell lines was quantified using qPCR. Data represent mean ± SEM. $n = 3$.

© 2018 The Authors
