# Supplementary figures and images for: FAM35A associates with REV7 and modulates DNA damage responses of normal and BRCA1‐defective cells
Source: EMBO J. 2018 May 22;37(12):e99543. doi: 10.15252/embj.201899543 (PMC6003645; doi:10.15252/embj.201899543)

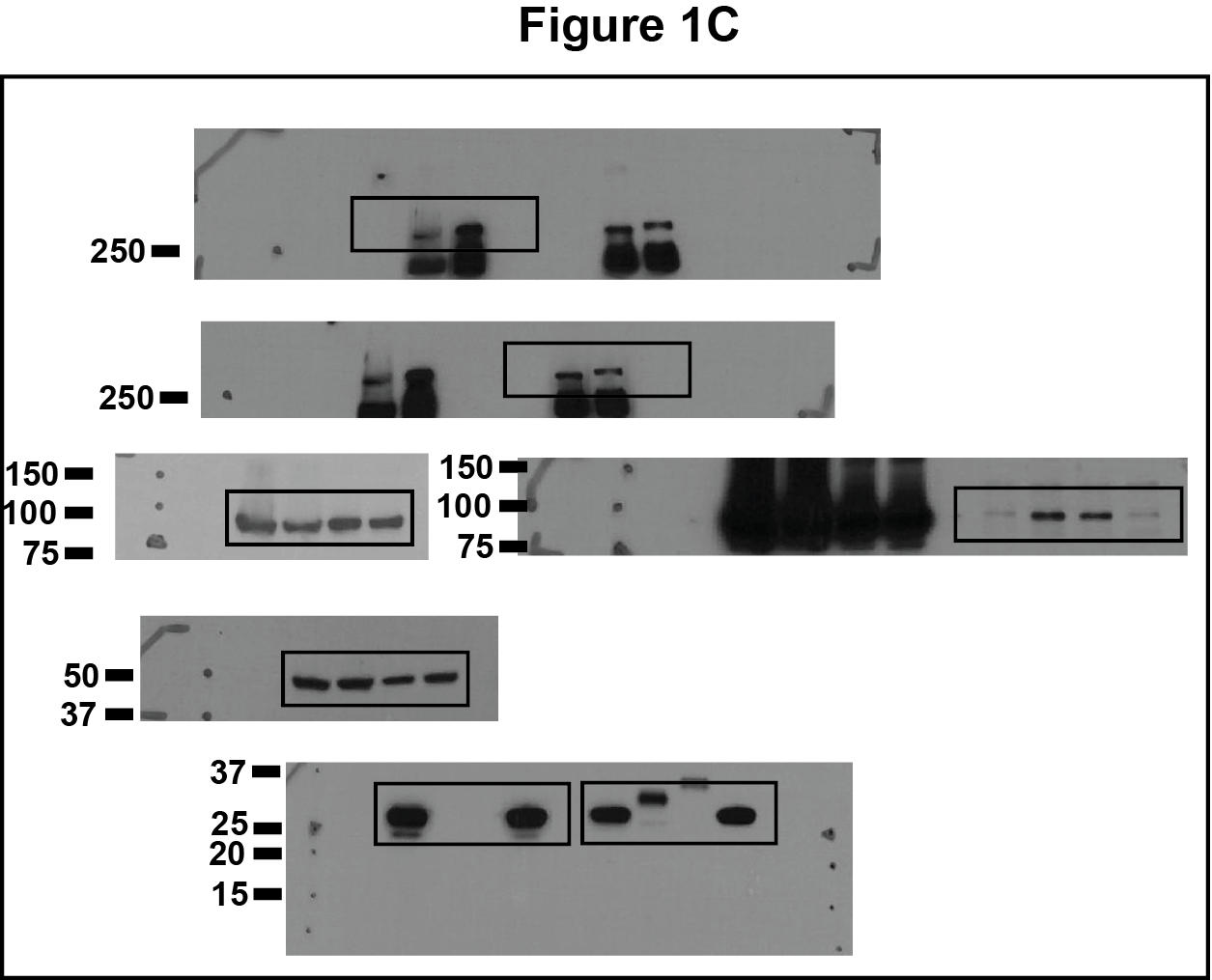

Supplement: Supplementary file 3 — Source Data for Figure 1 [file EMBJ-37-e99543-s002.tif]

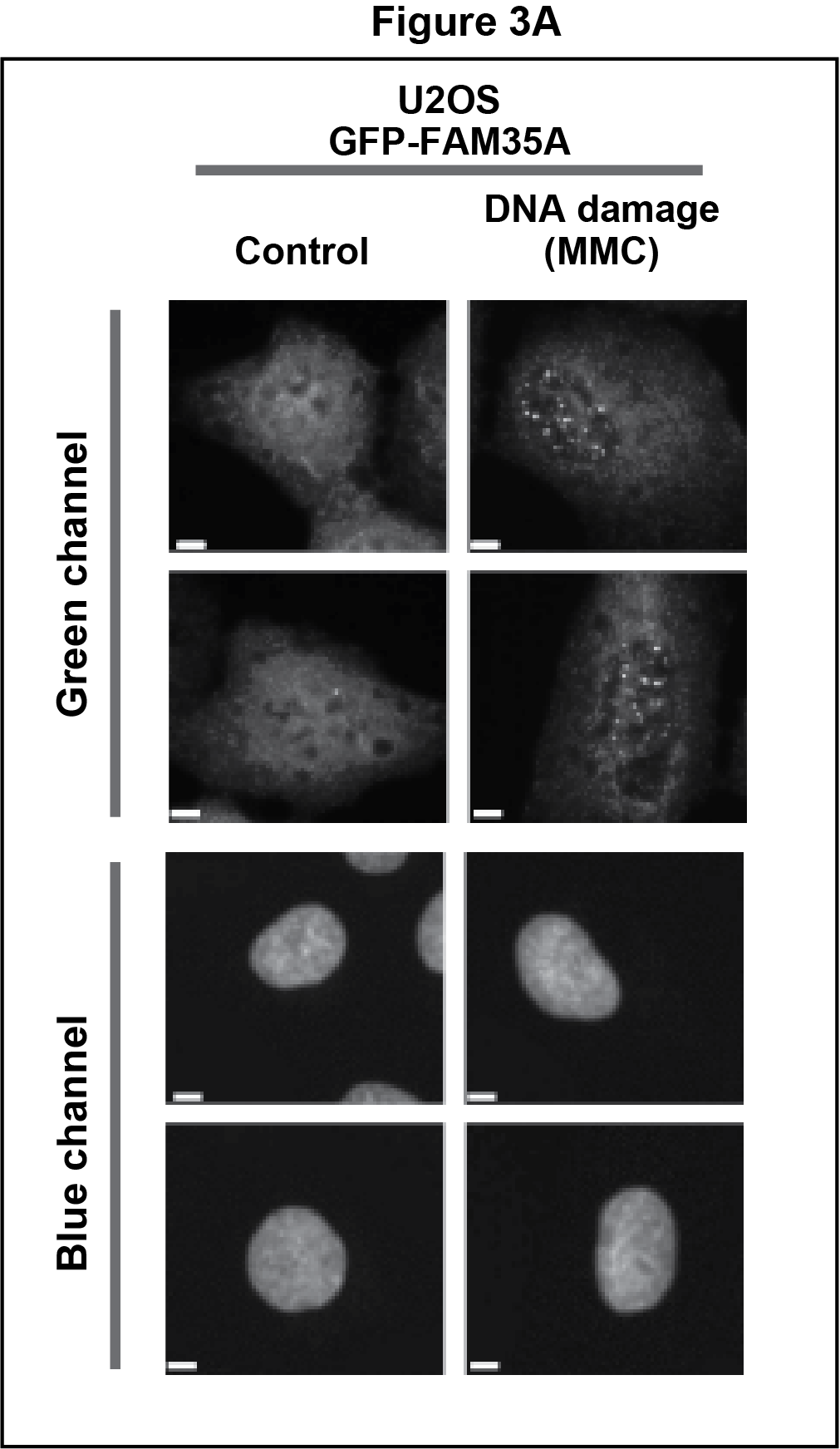

Supplement: Supplementary file 4 — Source Data for Figure 3 [file EMBJ-37-e99543-s003.tif]

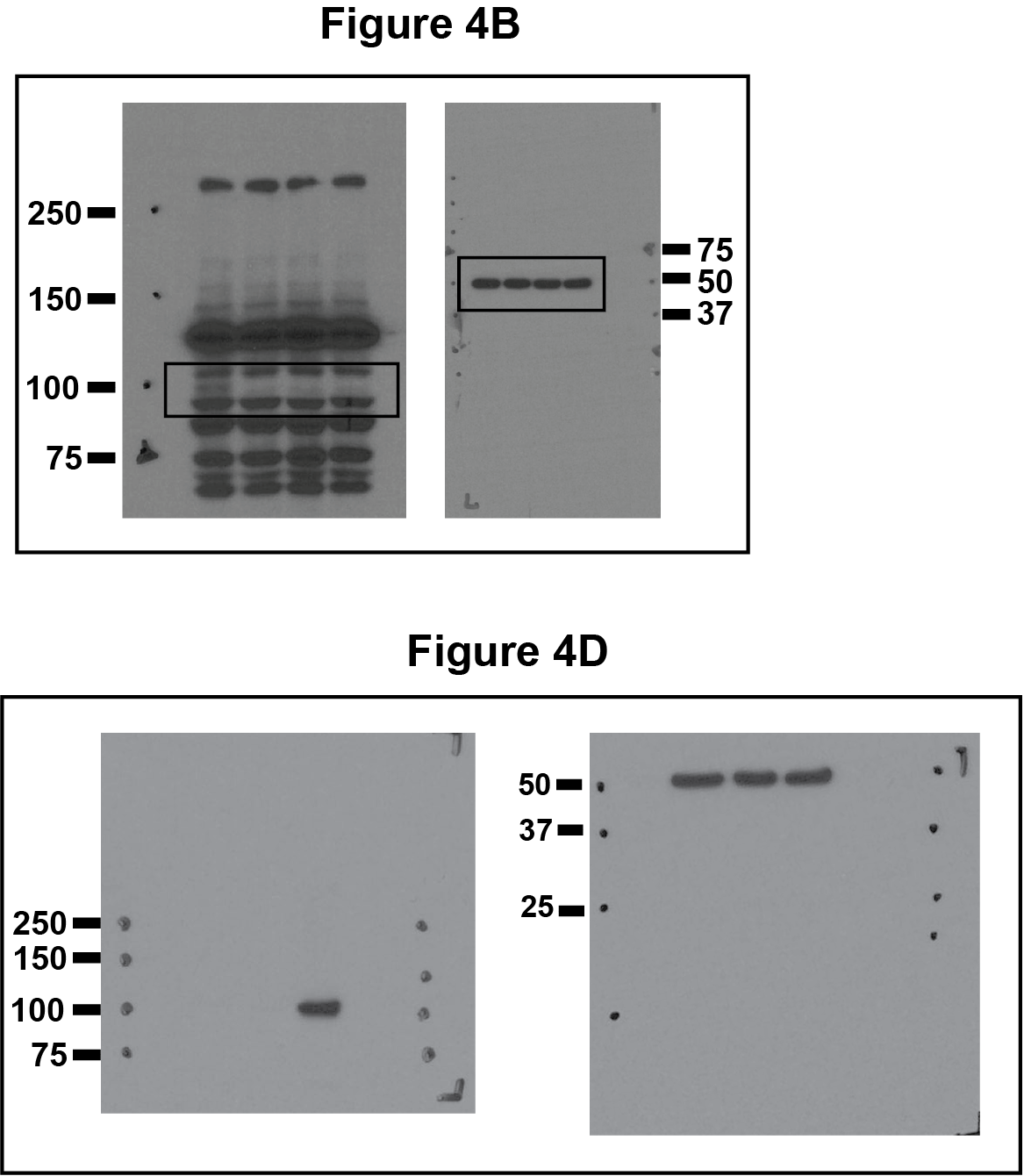

Supplement: Supplementary file 5 — Source Data for Figure 4 [file EMBJ-37-e99543-s004.tif]

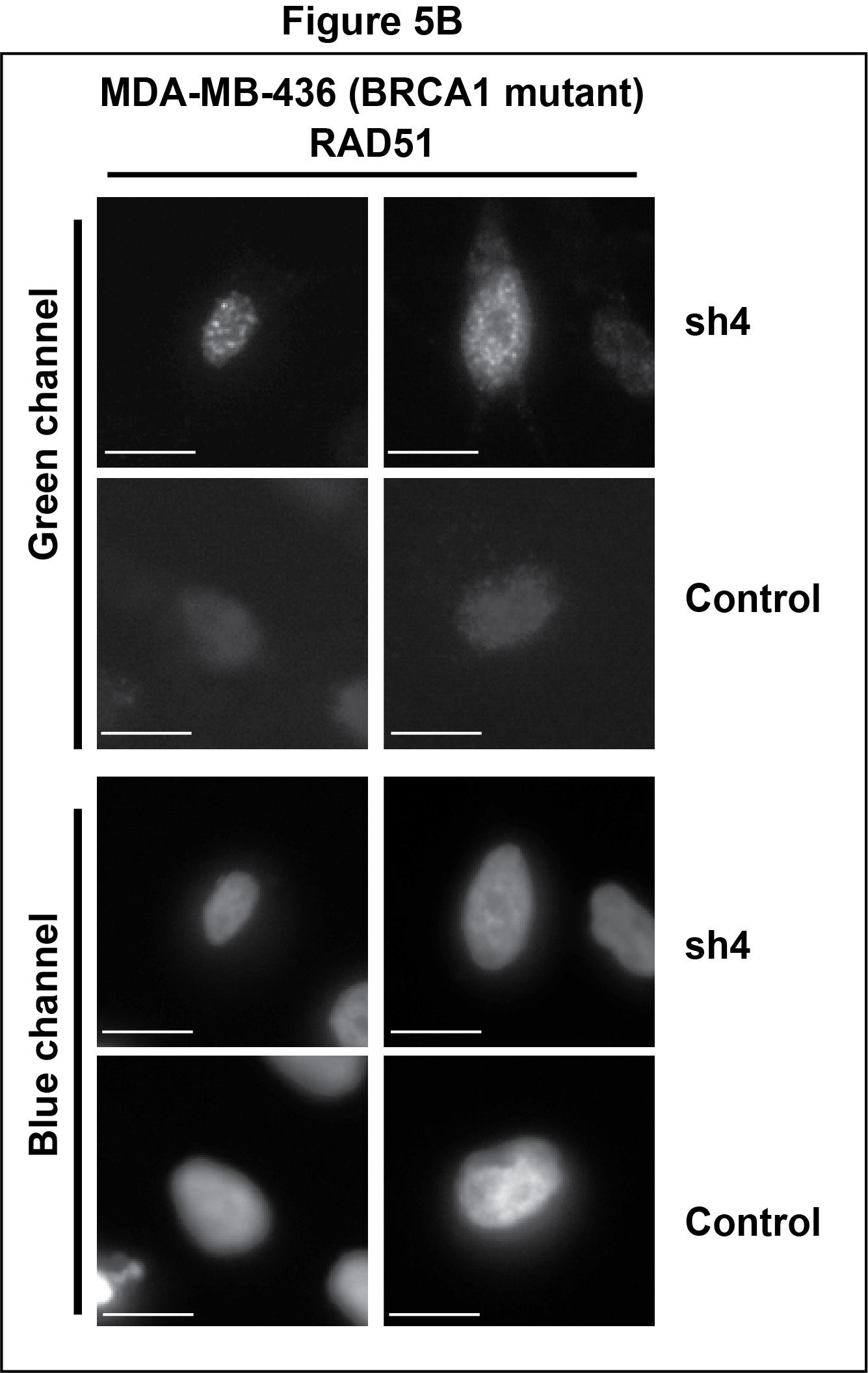

Supplement: Supplementary file 6 — Source Data for Figure 5 [file EMBJ-37-e99543-s005.tif]
